# Post-weaning social and cognitive performance of piglets raised pre-weaning either in a complex multi-suckling group housing system or in a conventional system with a crated sow

**DOI:** 10.1007/s10071-017-1110-x

**Published:** 2017-07-05

**Authors:** S. E. van Nieuwamerongen, M. Mendl, S. Held, N. M. Soede, J. E. Bolhuis

**Affiliations:** 1 Adaptation Physiology Group, Department of Animal Sciences, Wageningen University and Research, PO Box 338, 6700 AH Wageningen, The Netherlands; 2 Animal Welfare and Behaviour Group, School of Veterinary Sciences, University of Bristol, Bristol, UK

**Keywords:** Informed forager test, Social development, Cognitive development, Group housing, Multi-suckling system, Pigs

## Abstract

We studied the social and cognitive performance of piglets raised pre-weaning either in a conventional system with a sow in a farrowing crate (FC) or in a multi-suckling (MS) system in which 5 sows and their piglets could interact in a more physically enriched and spacious environment. After weaning at 4 weeks of age, 8 groups of 4 litter-mates per pre-weaning housing treatment were studied under equal and enriched post-weaning housing conditions. From each pen, one pair consisting of a dominant and a submissive pig was selected, based on a feed competition test (FCT) 2 weeks post-weaning. This pair was used in an informed forager test (IFT) which measured aspects of spatial learning and foraging strategies in a competitive context. During individual training, submissive (informed) pigs learned to remember a bait location in a testing arena with 8 buckets (the same bucket was baited in a search visit and a subsequent relocation visit), whereas dominant (non-informed) pigs always found the bait in a random bucket (search visits only). After learning their task, the informed pigs’ individual search visit was followed by a pairwise relocation visit in which they were accompanied by the non-informed pig. Effects of pre-weaning housing treatment were not distinctly present regarding the occurrence of aggression in the FCT and the learning performance during individual training in the IFT. During paired visits, informed and non-informed pigs changed their behaviour in response to being tested pairwise instead of individually, but MS and FC pigs showed few distinct behavioural differences.

## Introduction

Rearing conditions can have a great impact on an animal’s social and cognitive development. In conventional housing systems, piglets are reared in a farrowing pen with a crated sow, which provides an environment that is limited in stimuli that would promote piglet development: the sow is confined, which restricts sow–litter interaction and learning from the mother (Oostindjer et al. 2011b), piglets have no contact with other litters, the environment is generally barren with minimal enrichment material and no rooting substrate, and the pen has a relatively simple design. This is in large contrast to the environment that a pig would encounter under natural conditions. In the farrowing season, wild boar live in family groups consisting of several sows and their offspring, in a complex and enriched environment (Gundlach 1968; Meynhardt 1980). Given the opportunity, domestic pigs will form a similar social structure and display similar natural behaviours as their ancestors (Jensen 1986; Petersen et al. 1990; Stolba and Wood-Gush 1989). Hence, the social and physical environment in a conventional system restricts expression of natural behaviours, which may be important for the development of domestic pigs.

We have developed an alternative farrowing system that better resembles the natural situation, with more space and social and physical enrichment than in a conventional system (for a previous version of the system, see van Nieuwamerongen et al. 2015). This multi-suckling (MS) system houses five sows together with their piglets and has five farrowing pens connected to a communal area, which is divided into areas for resting, eating and dunging. This MS system has several properties that can affect piglets’ social and cognitive development: a spacious environment with enrichment, possibilities for interactions with multiple sows, and pre-weaning mingling with piglets from other litters. An increased space allowance and more complex pen design allow more possibilities for the development of a range of social skills, including avoidance and threatening behaviour (McGlone and Curtis 1985; Weng et al. 1998), and play behaviour, which is thought to be important for social development (Spinka et al. 2001). Compared with single-litter housing, pre-weaning contact with multiple litters has been shown to reduce aggression after weaning towards familiar pigs (Hessel et al. 2006) and unfamiliar pigs (Kanaan et al. 2012) and to stimulate quicker formation of a stable dominance hierarchy post-weaning (D’Eath 2005). The larger group size, resulting from pre-weaning mingling of sows and litters, can also affect social behaviour; pigs have been shown to display a lower level of aggression when housed in larger groups (Samarakone and Gonyou 2009).

Indeed, pigs reared in an MS system showed less aggression when mixed with unfamiliar pigs after weaning (Li and Wang 2011; Verdon et al. 2016) and established clearer dominance relationships among familiar pigs in a competitive situation, while also expressing less aggression (De Jonge et al. 1996). The latter study showed long-term effects of early social experiences, as differences between pigs from the different pre-weaning housing systems were found up until puberty. The larger group and more diverse group composition with multiple sows and litters in MS systems also provide more opportunities for social learning. In our MS system, social learning of eating behaviour is specifically stimulated by the use of a communal feeding area, where piglets can learn to eat solid food together with the sows and other piglets (see Oostindjer et al. 2011a).

As the MS system provides a more complex social and physical environment for the piglets, it might be expected that their cognitive development (which includes aspects of memory, learning, problem-solving and decision-making) is more stimulated in the MS system than in a conventional system. It has been hypothesised that one of the functions of cognition is to enable an animal to deal with environmental complexity, which includes aspects of both the social and physical (i.e. non-social) environment (Godfrey-Smith 2002). Specifically, the complexity of the social environment may be a driving force for the development of certain cognitive abilities, such as social learning (i.e. the acquisition of new skills, information or behaviour as result of interacting with other individuals) Arbilly et al. 2014; Croney and Newberry 2007; Godfrey-Smith 2002). Several studies have compared aspects of cognitive performance between pigs reared under physically enriched or barren conditions in spatial tasks. Sneddon et al. (2000) found that pigs reared in enriched pens learned a spatial task quicker than pigs reared in barren pens, although de Jong et al. (2000) found no such difference. Furthermore, enrichment generally improved aspects of short-term memory (e.g. working memory) and/or longer-term memory (e.g. reference memory; Bolhuis et al. 2013; de Jong et al. 2000; Grimberg-Henrici et al. 2016), although Jansen et al. (2009) found no difference between enriched and barren pigs in finding an alternative route to exit a maze and subsequently remembering the detour with a 1-day interval. Although not found consistently, it appears that an enriched environment may have beneficial effects on aspects of cognitive development.

Given that pigs reared in an MS system have experienced more social and physical environmental complexity than pigs reared in a conventional farrowing system, we hypothesised that MS pigs would be better prepared to deal with social and cognitive challenges later in life. We measured this using a feed competition test and a so-called informed forager test (IFT) after weaning. The IFT measures aspects of spatial learning and foraging strategies in a competitive context (Held et al. 2000). Two foraging roles have been described in several group foraging species: producers, which localise food sources autonomously, and scroungers, which eat from producers’ findings (Beauchamp and Giraldeau 1997; Giraldeau and Lefebvre 1986). When food is distributed in patches, as is the case for wild boar (Meynhardt 1980), the most dominant animals within a group can benefit from exploiting submissive producers, whereas the latter may best forage alone or find tactics to avoid exploitation (Held et al. 2000). Previous studies have shown that domestic pigs can flexibly adapt their behaviour to optimise their foraging strategy, depending on the type of food reward and the behaviour of other pigs (Held et al. 2000, 2002, 2005, 2010). In the IFT, pigs are directed to adopt a certain forager role, by training pairs consisting of a submissive ‘informed’ pig, which has knowledge about the location of a food reward in an arena with hidden buckets, and a dominant ‘non-informed’ pig, which is unaware of the reward’s location. In phase 1 of the IFT, both pigs are trained to be producers, as they have to find the reward individually. Informed pigs, however, learn that the location of the reward is always the same in two successive visits to the arena, whereas non-informed pigs learn that the reward is to be found in a non-predictable random location during each visit. In phase 2, the informed and non-informed pigs are tested in pairs, after the individual search visit of the informed pig. Thus, in phase 2, informed pigs are informed about the location of the reward, and non-informed pigs have the opportunity to switch to a scrounger role by following and displacing the informed pig from the reward. Subsequently, informed pigs may also alter their foraging strategy to minimise exploitation (Held et al. 2002).

We hypothesised that during the selection of pairs of dominant (non-informed) and submissive (informed) pigs, MS pairs would show less aggression and a clearer dominance relationship than the conventionally reared control pigs. Furthermore, we expected MS pigs to perform better than control pigs during the IFT. In other words, informed pigs would learn their task faster, both informed and non-informed pigs would demonstrate better working memory regarding food locations they have already visited and non-informed pigs would benefit more from the knowledge of informed pigs, if they have been raised in the MS system compared to control conditions.

## Materials and methods

### Animals and housing

The experiment was approved by the Animal Care and Use Committee of Wageningen University and Research. In total, 64 piglets (Tempo × Topigs 20) were studied, equally distributed over 2 successive batches. Before weaning, piglets were housed at the animal facilities of Swine Innovation Centre Sterksel, the Netherlands, either in a multi-suckling (MS) system consisting of 5 sows and their litters or with a sow in a conventional farrowing crate (FC). All sows were multi-parous, and allocation of the sows to the pre-weaning housing treatments was balanced for parity. In each batch, 4 healthy litter-mates from 4 litters were selected per system to participate in this study. Per litter, one light and one heavy piglet from both sexes were selected based on body weight 6 days before weaning (day −6). In addition, we took into account the relative weight difference per pre-weaning housing treatment. Before selection, mean litter weights at day −6 were 6.39 ± 0.30 kg in the MS system and 6.16 ± 0.34 kg in the FC system. Body weight of the selected piglets was 7.53 ± 0.23 kg for the ‘heavy’ MS piglets, 5.57 ± 0.23 kg for the ‘light’ MS piglets, 7.34 ± 0.23 kg for the ‘heavy’ FC piglets and 5.44 ± 0.20 kg for the ‘light’ FC piglets. After weaning at 27.1 ± 0.4 days of age, the piglets were transported to the research facility ‘Carus’ of Wageningen University and Research, the Netherlands. Post-weaning housing conditions (see below) were the same for all piglets.

### General pre-weaning management

Piglets were ear tagged and weighed within 24 h post-partum (p.p.). Litter sizes were standardised between 24 and 48 h p.p. according to the number of functional teats available per sow. Piglets were tail docked and received an iron injection within 4 days after birth. Male piglets were not castrated. Pre-starter creep feed (11.6 MJ of NE, 17.5% crude protein and 1.17% ileal digestible lysine) was provided to the piglets in round feeders (diameter 28 cm) twice daily from day 12. On day 21–22, pre-starter feed was gradually mixed with weaner feed (9.9 MJ of NE, 16.0% crude protein and 0.99% ileal digestible lysine), and after day 22 only weaner feed was provided. Water was available ad libitum. Animal health was checked twice daily.

### Pre-weaning housing

#### Multi-suckling system

**Fig. 1:**
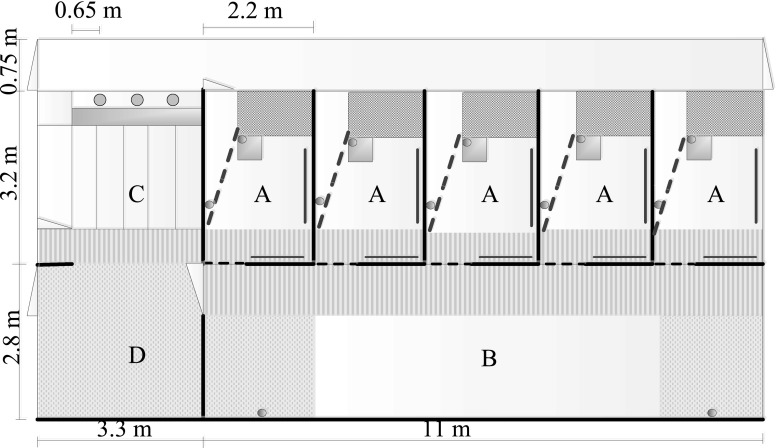
Layout of the multi-suckling system. The system has 5 farrowing pens (*A*—with piglet nest, sow feeder, drinkers and anti-crushing devices) connected to a communal area with a lying area (*B*—with 2 drinkers), feeding area (*C*—with 5 sow feeding places surrounded by a piglet area with piglet feeders) and a dunging area (*D*)

MS housing consisted of 5 farrowing pens connected to a communal area (Fig. 1). Sows could access all areas from the moment of entry in the system. The farrowing pens were 3.2 × 2.2 m each and contained a feed trough for the sow, a water nipple for the piglets, and a covered piglet nest of 0.7 × 1.6 m with heated solid flooring. The floor in the rest of the pen consisted of solid concrete and concrete slats. Five hessian sacks were provided per pen as nesting material. From day 2 p.p. onwards, two handfuls of long-stemmed straw were provided daily per farrowing pen. The adjacent communal area was divided in an area for feeding (solid concrete and metal slats), defecating/urinating (metal slats) and lying (solid concrete and metal slats). Five hessian sacks and five ropes were attached to the partitions surrounding the resting area and were replaced if needed. The communal feeding area contained five feeding places for the sows separated by horizontal metal bars, and a surrounding area with piglet feeders accessible to only the piglets. Each individual sow was locked in her own farrowing pen daily between 16:30 and 7:30 until farrowing of that particular sow had ended. From that moment onwards, sows could freely access all farrowing pens and communal areas. Piglets were given access to the whole system at a mean age of 7.9 ± 0.3 days. This was achieved by removing a 31-cm-high piglet barrier at the entrance from each farrowing pen. In addition, a ‘piglet door’ (0.4 × 0.3 m) was removed from the front wall of each farrowing pen, to provide piglets more space to move in and out of the farrowing pens. The sows were floor fed twice daily in the communal area, and piglets could eat together with the sows from the sow feed and from creep feed provided in the piglet feeders. Sows were fed in their own farrowing pen only in the afternoons before farrowing and in the first days after farrowing in case the sows did not leave their pen to eat in the communal area.

#### Conventional system

Each FC litter was kept in a pen of 2.4 × 1.8 m, which contained a farrowing crate of 2.0 × 0.7 m for the sow. The pens were situated in farrowing units containing 12 pens each. The floor consisted of metal slats within the crate, a solid floor of 1.2 × 0.3 m with a heat lamp for the piglets and plastic slats in the remaining area. The sow had a feed trough and a drinking nipple available. The piglets had access to a separate drinking nipple, and creep feed was provided in a round feeder located in the corner at the posterior side of the sow. One day prior to expected farrowing, sows received a hessian sack as nesting material. During the whole pre-weaning period, a plastic roll was available for both sow and piglets and a chain with a wooden block was available as enrichment for the sow.

### Post-weaning housing

After weaning, 32 piglets per batch were transported (for about 1.5 h) to their new housing facilities. During transport, the 16 MS piglets were penned in one group, whereas the 16 FC piglets were penned in groups of 4 litter-mates. After arrival, MS and FC litters were equally divided over 2 adjacent rooms, where the 4 pens in one room were filled alternatingly with piglets from MS and FC litters. Each pen (2.7 × 3.6 m) housed 4 litter-mates and was bedded with 550 L of sawdust, 10 kg of straw and 90 L of peat on a solid floor. On a daily basis, soiled bedding was removed from the pens and 70 L of fresh sawdust and 1 kg of straw were added. Fresh peat was added on a weekly basis (45 L). A hessian sack, a rope and a chain with bolts were available as further enrichment. The same weaner feed that was provided in the late pre-weaning phase was offered ad libitum in a feeder with 4 eating places. Two weeks after weaning, a starter diet (9.6 MJ of NE, 17.3% crude protein and 1.04% ileal digestible lysine) was provided in a new feeder with one eating place. A grower diet was provided from 5 weeks post-weaning onwards (9.6 MJ of NE, 16.3% crude protein and 0.93% ileal digestible lysine). Water was continuously available from a drinking nipple. Animal health was checked twice daily. All pigs were marked with a number on their back using stock marker spray to allow individual identification.

### Behaviour tests

#### Feed competition test

The feed competition test (FCT) took place on day 14 and 15 post-weaning. Piglets were feed deprived by removing the feeders from the pens in the afternoons before the tests (around 16:30). All 4 possible combinations of a ‘heavy’ versus a ‘light’ piglet were tested per pen. Tests were divided over 2 mornings, and 2 pairs of piglets were tested per pen before proceeding to the next pen. Each piglet was tested only once per morning. The order of testing pens was the same for both testing days and was balanced for pre-weaning housing treatment. Before a pair was tested, all 4 piglets were removed from their pen and led into the corridor adjacent to the pens. A bucket attached on top of 2 wooden boards arranged in a cross (to prevent piglets from knocking over the bucket) was placed where the feeder was normally located. The boards were covered with bedding material to prevent piglets from slipping on the boards. The bucket was filled with 100 g of the pigs’ standard feed. The pair to be tested was separated from the other 2 litter-mates using a board and was led back into their pen. The test started when the first pig had completely entered the pen and lasted for 4 min. The behaviours listed in Table 1 were recorded by 2 trained observers (one fixed observer scored bucket access for both batches, and one fixed observed scored all agonistic interactions for both batches). After the test, all 4 piglets were led back into their pen. The feeders were put back in the pens after all tests of one morning were completed. Based on the performance in the FCT, one pair per pen was selected to participate in the informed forager test, with the dominant pig being trained as the non-informed pig and the submissive pig being trained as the informed pig. The FCT was repeated at the end of batch 2 for pairs used in the IFT only (day 79, with 400 g of feed per bucket), to check the stability of the dominance relationships.

**Table 1:** Ethogram used in the feed competition test and informed forager test (based on Schouten (1985) and Held et al. (2000))

Measurement	Description
Bucket access (duration)	Piglet has its head in the bucket and has exclusive access to the bucket
Head knock (frequency)	Piglet gives a single horizontal or vertical knock with the head or a forward thrust with the snout towards the other piglet, without biting
Bite (frequency)	Piglet gives a single bite (snapping jaws) at the other piglet. Can be performed while giving a head knock
Push (frequency)	Piglet exerts force with the body to the other piglet’s body (without displacing the other piglet)
Displacement (frequency)	Piglet gains exclusive access to the bucket by pushing the other piglet away from the bucket while the other piglet had bucket access

#### Informed forager test

**Fig. 2:**
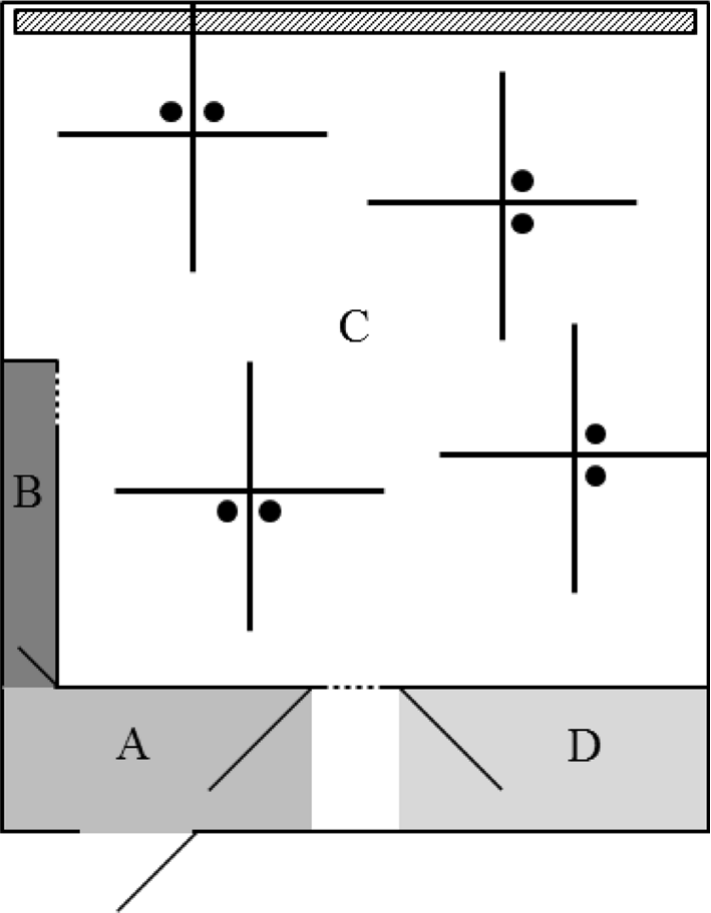
Layout of the testing room (7.4 × 6.3 m) used for the informed forager test. *A* = the area where the pigs entered the testing room, *B* = the start box, *C* = the testing arena, which contained 4 crosses (60 cm high) with 2 buckets (*black circles*) per cross and *D* = the waiting area for the litter-mates that did not participate in the test, containing 2 toys and a hessian sack. *Dotted lines* represent guillotine doors, and *angled solid lines* represent hinged doors. The *dashed area* in the back represents a drainage area

The informed forager test (IFT) was carried out from 5 to 15 weeks of age and consisted of 2 phases, based on Held et al. (2000). In short, after a habituation period, individual piglets were trained in phase 1 to locate one baited bucket in a testing arena (see Fig. 2). Informed pigs were the submissive pigs within each pair and were allowed to search for the bait in the testing arena twice within a trial (i.e. one search visit and one relocation visit), with the bait located in the same bucket in both visits of the trial. Informed pigs were thus trained to remember the specific location of a baited bucket after a search visit and use this information in a subsequent relocation visit. The non-informed pigs were the dominant pigs within each pair and were trained to search for the baited bucket, without having prior knowledge about the location of the bait in the testing arena (i.e. non-informed pigs had only search visits). In phase 2, informed pigs were accompanied by their non-informed pen mate during the informed pig’s relocation visit. In both phases of the IFT, one trial for a non-informed pig consisted of a search visit, and one trial for an informed pig consisted of a search visit followed immediately by a relocation visit.

##### Habituation to the informed forager test

Before starting the IFT, the piglets were gradually habituated to elements of the test over a course of 13 days when they were 5–7 weeks old. The piglets were sequentially habituated to: being in the presence of people in their home pen, touching buckets with bait and eating the bait in their home pen, visiting the testing room with all 4 pen mates and with all 8 buckets baited, visiting the testing room individually with 8 baited buckets, visiting the testing room individually with 4 baited buckets, visiting the testing room individually with 2 baited buckets and, finally, visiting the testing room individually with 2 baited buckets and all buckets covered with chopped straw. Piglets were exposed to the testing room with only one baited bucket for the first time during testing.

##### General procedure for informed forager test phases 1 and 2

The IFT took place between 8 and 15 weeks of age. Two trials were run daily from Monday up until Friday between 8:30 and 13:30. Pigs were feed deprived by closing the feeders around 16:30 on the day before each testing day. The metal buckets in the testing room all contained a removable metal grid, creating a double bottom under which bait (4 chocolate raisins) could be placed. Before pigs entered the testing arena, bait was placed under the double bottom of each bucket, which equalised the odour cues from each bucket, but prevented pigs from accessing the bait. All double bottoms were covered with chopped straw (to hide bait from direct sight and increase searching time), and one bucket was filled with bait that was placed within the chopped straw and was thus accessible to the pigs. The location of this baited bucket was randomised for every subsequent trial. For each trial, the 4 pigs from one pen were guided to the testing room. The test started when the pig (or first pig in paired visits) had completely entered the arena, and the test ended when the pig (or last eating pig in paired visits) had lifted its head from the baited bucket. An auditory cue and the opening of the exit door (between A and D in Fig. 2) then followed, after which the pig(s) left the testing arena. The maximum testing duration per visit was 3 min. If the bait had not been found within this time, an experimenter stepped in the testing arena and showed the location of the bait, by walking to the baited bucket and enticing the pig to follow. When the pig(s) had left the testing arena, leftover bait was quantified and removed, the straw of the bucket that was baited was replaced and new bait was placed. Between visits, faeces and urine were removed from the testing arena if needed. After testing both the informed and non-informed pig, they returned together with their 2 pen mates to their home pen where they received 4 handfuls of feed on the floor after each trial. Before bringing pigs from the next pen to be tested to the testing room, faeces and urine were removed from the testing arena, start box and waiting area, if needed. All pens were tested during the first series of trials, before starting the second series of trials. Pens were tested in the same order during the first and second series of trials. The testing order per day was randomised and balanced for pre-weaning housing treatment. After the pigs from a pen finished the second trial, the feeder in their home pen was opened again. After each testing day, the whole testing room was cleaned with water and all-purpose cleaner.

During phase 2 in batch 1, few interactions between the informed and non-informed pigs occurred, and therefore, the procedure and the bait used for the paired visits were adjusted for batch 2. The bait was changed to 4 chocolate raisins and 25 g of feed in the individual visits (this was also the bait that was placed under the double bottoms) and 6 chocolate raisins and 50 g of feed in the paired visits. In addition, non-informed pigs were given more opportunity to discover that there was still a baited bucket to be found in phase 2 (and not only in phase 1). This was done by using a minimum testing duration of 2 min (while still maintaining the maximum testing time of 3 min), only in case the informed pig found the bait without the non-informed pig being present near the baited bucket (i.e. in the imaginary diamond shape that was formed by the crosses, see Fig. 2).

##### Informed forager test phase 1: individual training

The informed pig was guided to the start box while its pen mates remained in the waiting area. As soon as the door to the testing arena was opened, the informed pig was allowed to search for the bait. After finding the bait, the informed pig was guided via area A (see Fig. 2) to the start box again for a relocation visit in which the bait was placed in the same bucket. After completing the relocation visit, the informed pig was guided to the waiting area. Thereafter, the non-informed pig was released into the testing arena and was allowed to search for the bait that was placed in a different randomised location. The frequency, duration and latency of visits to all buckets were scored by one trained observer. The difference between search and relocation visits in the number of bucket visits, revisits to buckets already inspected and the latency to find the bait was used to indicate whether informed pigs learned their task. Moreover, perseverance errors were scored to test the tendency of informed pigs to search for the bait in the same location in the two successive trials of one day (as they were trained to relocate the same baited bucket within a trial and search for the bait in a different location in the next trial). A perseverance error occurred on days where an informed pig, during the second trial of the day, first visited the bucket that was baited in the first trial of that day. Perseverance errors were not taken into account for non-informed pigs, as they always found the bait in a random location. For both informed and non-informed pigs, revisits to buckets were assessed as a measure of impaired working memory (van der Staay et al. 2012). The informed and non-informed pig from a pen proceeded to phase 2 when the informed pig reached the criterion of visiting a maximum of 2 unbaited buckets during the relocation visits in at least 6 out of 8 consecutive trials (Held et al. 2005, 2010).

##### Informed forager test phase 2: pairwise testing

After individual training, informed pigs were tested together with their non-informed pen mate. The search visit of the informed pigs was executed as in phase 1. During the relocation visit, however, the informed pig was accompanied by its non-informed pen mate. The frequency, duration and latency of visits to all buckets and the interactions between the 2 pigs were scored by 2 observers, with each observer scoring one of the 2 pigs (see Table 1). In total, 4 observers were involved in behavioural observations over 2 batches. All observers were trained prior to the start of data collection, and allocation of observers to an animal to be observed was balanced for pre-weaning housing treatment, litter, pig status (informed or non-informed) and testing day. The differences between phases 1 and 2 in the number of bucket visits, revisits and the latency to reach the first bucket and the baited bucket were used as indicators of the informed and non-informed pigs’ response to individual visits versus paired visits. Moreover, the number of ‘I–NI visits’, where the non-informed pig visited the bucket that the informed pig investigated immediately before, was used to determine in which trials ‘close following’ of the informed pig by the non-informed pig occurred. The order of bucket visits was used as a criterion for I–NI visits (without restriction by a maximum time interval between bucket visits), consistent with the protocol of Held et al. (2000). Trials with ‘close following’ were those in which the number of I–NI visits divided by the total number of bucket visits of the non-informed pig was ≥0.5 (Held et al. 2000) and were used to indicate whether non-informed pigs made use of the information of the informed pigs (in case a non-informed pig did not visit any buckets within a trial, the proportion of I–NI visits was set to zero). The number of displacements of the non-informed pig towards the informed pig was used to indicate whether the non-informed pigs exploited the informed pigs (Held et al. 2000).

### Statistical analysis

Data were analysed with SAS 9.2 (SAS Inst. Inc., Cary, NC). Residuals were checked for normality, and variables were transformed with a square root if needed. Results are presented as mean ± SEM. *p* values below 0.05 were considered statistically significant.

#### Feed competition test

Variables of the FCT were analysed using mixed models with pre-weaning housing treatment and batch as fixed effects, and a random effect of pen. The relative weight difference within the pair influenced total frequency of aggressive behaviours and tended to influence the number of displacements and was therefore added to the model as a covariate.

For the pairs selected per pen to participate in the IFT, differences in behaviour and body weight between non-informed and informed pigs, and the effect of pre-weaning housing on these differences, were analysed in a mixed model with pre-weaning housing treatment, status (informed or non-informed), housing treatment × status interaction, and batch as fixed effects, and a random effect of pen.

#### Informed forager test phase 1

The number of trials run during phase 1 ranged from 18 to 31 per pen. As all pens completed at least 18 trials in phase 1, these first 18 trials per pen were analysed for treatment effects during individual training (when including all trials of phase 1, instead of only the first 18, similar results were found). During 24.2% of all visits in these 18 trials, the maximum testing time elapsed and pigs were guided to the baited bucket. The occurrence of these guided visits did not differ significantly between MS (27.3%) and FC (21.1%) pigs and were included in analyses. Results were similar whether these visits were included or not, unless indicated otherwise in the results.

The difference in number of bucket visits, revisits and latency to reach the baited bucket between search and relocation visits of the informed pigs, and the effect of treatment on these differences were analysed using mixed models with pre-weaning housing treatment, visit type (search or relocation visit), housing treatment × visit type interaction and batch. Repeated observations on the same individuals were taken into account by including a repeated effect of visit type at pen level.

To investigate the learning curve of the informed pigs during relocation visits over time, the number of bucket visits, revisits and latency to reach the baited bucket was analysed using the 18 trials averaged per testing day (i.e. 9 testing days were taken into account in the analyses). Mixed models were used, including pre-weaning housing treatment, testing day, housing treatment × day interaction and batch. Repeated observations on the same individuals were taken into account by including a repeated effect of testing day at pen level.

The percentage of days during which a perseverance error occurred and the number of trials needed to reach the criterion to proceed to phase 2 were analysed with mixed models including pre-weaning housing treatment and batch as fixed effects. The same model was used to analyse the number of bucket visits, revisits, the latency to reach the first bucket, and the latency to reach the baited bucket for the non-informed pigs.

#### Comparisons between informed forager test phases 1 and 2

The number of trials run for phase 2 varied between 28 and 41 trials per pen. For the comparison between both phases of the IFT, the last 8 trials of phase 1 of a particular pair (in which informed pigs had all reached the criterion) and the first 28 trials of phase 2 of a particular pair were analysed. The number of bucket visits, revisits and latency to reach the first bucket and the baited bucket was analysed with mixed models including pre-weaning housing treatment, phase, batch, and their 2-way and 3-way interactions. Batch was included in the interactions because the bait and testing procedure during phase 2 differed in batch 1 and batch 2. Repeated observations were taken into account by including a repeated effect of phase at pen level.

#### Informed forager test phase 2

The proportion of I–NI visits and closely followed trials were analysed with generalised linear mixed models, including pre-weaning housing treatment, batch and their interaction. The percentage of trials in which a pig ate from the bait was analysed with a generalised linear mixed model including status (informed vs. non-informed) as a fixed effect, and pen within treatment and batch as a random effect. The generalised linear mixed models had a binomial distribution and logit link function. The occurrence of displacements was analysed with a Fisher’s exact test, as MS pairs did not show any displacements in batch 1. Because no interactions can be analysed in the Fisher’s exact test, both pre-weaning housing treatments were compared within each batch, and both batches were compared within each pre-weaning housing treatment. Relations between the percentage of trials in which informed and non-informed pigs ate from the bait and the behaviour of both pigs were analysed with Spearman correlations, using averages per pen over the first 28 trials of phase 2.

## Results

### Feed competition test

When considering all heavy versus light pig combinations (4 per pen), the total frequency of aggressive behaviours (i.e. head knocks, bites, pushes, displacements, and the frequency of these behaviours summed together) and the absolute difference in aggression between the 2 pigs tested were not affected by pre-weaning housing treatment (data not shown). Also, the overall duration of bucket access, the difference within a dyad in the duration of bucket access and the total number of bucket access bouts did not differ between MS and FC pigs. However, the difference within dyads in the number of bucket access bouts was smaller for MS pigs than for FC pigs (0.8 ± 0.2 vs. 1.8 ± 0.3, *F*_1,13_ = 5.91, *p* < 0.05).

From each pen, one dyad was selected to represent a dominant and a submissive pig. From these selected pairs, the non-informed pig was on average 27.4% heavier than the informed pig (12.1 ± 0.5 vs. 9.5 ± 0.4 kg, *F*_1,14_ = 139.09, *p* < 0.0001) and the non-informed pig successfully displaced the informed pig to get to the feed 3.3 times more often (3.9 ± 0.5 vs. 1.2 ± 0.4, *F*_1,14_ = 72.31, *p* < 0.0001). Moreover, during the 4-min test, the non-informed pigs had 2.4 times longer access to the bucket with feed than the informed pigs (156.6 ± 10.2 vs. 65.8 ± 6.5 s, *F*_1,14_ = 53.16, *p* < 0.0001). These differences between non-informed and informed pigs were not affected by pre-weaning housing conditions. Additionally, FC non-informed pigs performed more head knocks, bites and pushes than FC informed pigs (13.9 ± 2.7 vs. 6.6 ± 1.5), whereas status did not affect the frequency of these aggressive behaviours among MS pairs (MS informed: 8.5 ± 1.9, MS non-informed 8.5 ± 2.0, treatment × status interaction, *F*_1,14_ = 4.72, *p* < 0.05).

Concerning the FCT repeated for the 8 pairs in batch 2; on day 79, non-informed pigs were still heavier than their informed partners (71.9 ± 1.9 vs. 63.2 ± 2.1 kg, *F*_1,6_ = 49.78, *p* < 0.001), successfully displaced the informed pig more frequently (1.6 ± 0.3 vs. 0.5 ± 0.4, *F*_1,6_ = 9.00, *p* < 0.05), but did not have significantly longer access to the bucket with feed (110.5 ± 13.5 vs. 72.6 ± 14.0 s, *F*_1,6_ = 3.57, *p* = 0.11).

### Informed forager test phase 1

#### Informed pigs

In phase 1 of the IFT, informed pigs were trained to remember the specific location of a baited bucket after a search visit and a subsequent relocation visit. The retention interval, i.e. the time between the start of the search visit and of the relocation visit, was 4 ± 1 min (average ± SD). The effects of pre-weaning housing conditions and differences in performance between search and relocation visits for informed pigs over the first 9 testing days (18 trials) are summarised in Table 2. During the relocation visits, informed pigs visited fewer buckets (3.4 ± 0.2 vs. 5.4 ± 0.2), had fewer revisits to buckets that were already investigated (0.2 ± 0.04 vs. 0.8 ± 0.09) and had a shorter latency to reach the baited bucket than in the search visits (59.7 ± 4.4 vs. 97.8 ± 7.0 s). Pre-weaning housing did not affect these differences between search and relocation visits of the informed pigs.

**Table 2:** Performance during the first 9 testing days (18 trials) of phase 1 of the informed forager test

Variable	Search visit		Relocation visit		*p* values		
	MS	FC	MS	FC	Housing	Visit	Housing × visit
*Informed pig*							
Number of bucket visits	5.6 ± 0.4	5.2 ± 0.3	3.7 ± 0.3	3.1 ± 0.2	0.12	<0.0001	0.77
Number of revisits	0.7 ± 0.1	0.8 ± 0.2	0.3 ± 0.1	0.2 ± 0.04	0.48	<0.0001	0.52
Latency to baited bucket (s)	98.2 ± 10.7	97.5 ± 9.7	64.1 ± 6.6	55.4 ± 5.8	0.59	0.0006	0.65
*Non*-*informed pig*							
Number of bucket visits	4.1 ± 0.6	4.8 ± 0.3	–	–	0.07	–	–
Number of revisits	0.3 ± 0.1	0.3 ± 0.05	–	–	0.81	–	–
Latency to baited bucket (s)	107.0 ± 15.4	76.0 ± 6.8	–	–	0.008	–	–

Informed pigs had to find a baited bucket in the ‘search’ visit and relocate the same baited bucket during the ‘relocation’ visit. Non-informed pigs had only ‘search’ visits in which they had to find a randomly located baited bucket. Pigs were housed pre-weaning either in a multi-suckling (MS) system with 5 sows and their piglets, or with a sow housed in a farrowing crate (FC) and only litter-mates

**Fig. 3:**
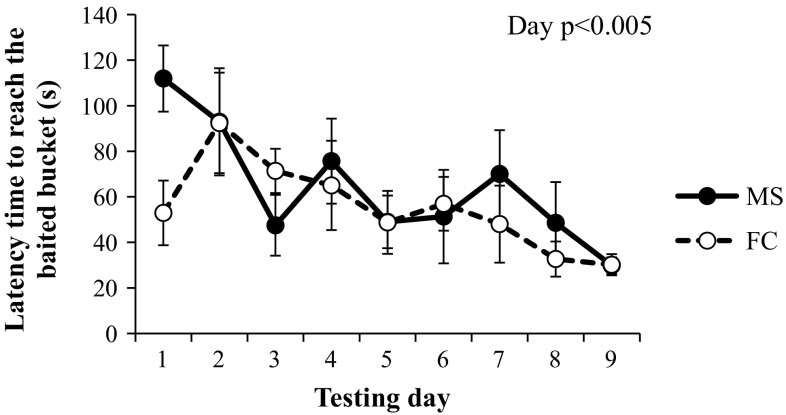
Latency to reach the baited bucket for informed pigs during the relocation visits of the first 9 testing days (18 trials) of phase 1 of the informed forager test. The informed pigs had to find a baited bucket in the ‘search’ visit and relocate the same baited bucket during the ‘relocation’ visit. Pigs were housed pre-weaning either in a multi-suckling (MS) system with 5 sows and their piglets, or with a sow housed in a farrowing crate (FC) and only litter-mates

The percentage of days on which informed pigs made a perseverance error (MS: 23.8 ± 4.7, FC: 23.4 ± 3.1) and the number of trials needed to reach the criterion to proceed to phase 2 of the IFT (MS: 20.5 ± 2.8, range 11–31; FC: 17.4 ± 2.0, range 8–25) did not differ between pre-weaning housing treatments. During relocation visits of informed pigs, the latency to reach the baited bucket decreased over the first 9 testing days (Fig. 3), but there was no day effect on the number of bucket visits and revisits (data not shown). When omitting the guided visits (i.e. 24.2% of all visits in the first 18 trials), the day effect on latency to reach the bait became non-significant.

#### Non-informed pigs

Non-informed MS pigs tended to visit fewer buckets and had a longer latency to reach the baited bucket than non-informed FC pigs during the search visits over the first 9 test days (18 trials). The number of revisits did not differ between non-informed MS and FC pigs (Table 2).

### Informed forager test phase 2

#### Informed pigs

During the paired visits in phase 2, informed pigs tended to have a longer latency to visit the first bucket (15.3 ± 1.5 vs. 11.0 ± 1.2 s), visited fewer buckets (1.9 ± 0.1 vs. 2.5 ± 0.1) and tended to have fewer revisits (0.01 ± 0.01 vs. 0.11 ± 0.05) than during the individual visits in phase 1 (Table 3). Additionally, informed MS pigs visited more buckets than informed FC pigs during phases 1 and 2 combined (2.4 ± 0.1 vs. 2.0 ± 0.1).

**Table 3:** Performance in the last 8 trials of phase 1 (individual visits) and the first 28 trials of phase 2 (paired visits) of the informed forager test^a^ for different housing systems^b^

Variable	Batch 1				Batch 1			
	Phase 1—individual visits		Phase 2—individual visits		Phase 1—individual visits		Phase 2—individual visits	
	MS	FC	MS	FC	MS	FC	MS	FC
*Informed pig*								
Latency to first bucket (s)	13.8 ± 3.0	10.0 ± 1.9	14.7 ± 3.4	19.0 ± 2.9	8.4 ± 1.4	11.9 ± 3.3	13.5 ± 2.0	14.1 ± 3.6
Number of bucket visits	2.7 ± 0.3	2.1 ± 0.3	2.1 ± 0.2	1.8 ± 0.1	2.7 ± 0.4	2.5 ± 0.1	2.2 ± 0.2	1.8 ± 0.1
Number of revisits	0.00 ± 0.00	0.13 ± 0.09	0.02 ± 0.01	0.00 ± 0.00	0.22 ± 0.15	0.09 ± 0.06	0.03 ± 0.02	0.00 ± 0.00
Latency to baited bucket (s)	33.3 ± 4.7	27.2 ± 5.4	32.6 ± 8.0	31.0 ± 4.9	30.6 ± 5.2	35.2 ± 5.7	36.0 ± 5.8	27.7 ± 2.9
*Non-informed pig*								
Latency to first bucket (s)	5.2 ± 0.9	6.1 ± 0.5	13.9 ± 2.3	18.4 ± 4.0	16.8 ± 6.8	5.7 ± 0.8	23.3 ± 4.5	15.3 ± 1.7
Number of bucket visits	5.3 ± 0.4	4.4 ± 0.7	2.1 ± 0.5	2.2 ± 0.4	4.2 ± 0.5	4.4 ± 0.3	1.9 ± 0.2	2.8 ± 0.2
Number of revisits	0.09 ± 0.06	0.44 ± 0.16	0.04 ± 0.03	0.04 ± 0.01	0.22 ± 0.11	0.13 ± 0.07	0.03 ± 0.02	0.03 ± 0.02
Latency to baited bucket (s)	69.0 ± 18.9	57.4 ± 16.8	54.7 ± 14.8	61.4 ± 12.3	96.3 ± 15.1	62.2 ± 15.4	61.5 ± 12.8	43.6 ± 1.4
Proportion of I–NI visits	–	–	0.09 ± 0.02	0.12 ± 0.05	–	–	0.29 ± 0.03	0.25 ± 0.07
Proportion of closely followed trials	–	–	0.08 ± 0.02	0.10 ± 0.03	–	–	0.33 ± 0.04	0.27 ± 0.10
Proportion of trials with diaplacements	–	–	0/28	14/28	–	–	21/28	12/28

Variable	Housing	Phase	Batch	Housing × phase	Housing × Phase	Phase × batch	Housing × phase × batch
*Informed pig*							
Latency to first bucket (s)	0.56	0.05	0.28	0.52	0.65	0.75	0.19
Number of bucket visits	0.03	0.005	0.45	0.83	0.74	0.70	0.42
Number of revisits	0.80	0.07	0.30	0.26	0.23	0.36	0.28
Latency to baited bucket (s)	0.47	0.95	0.73	0.60	0.79	0.74	0.28
*Non-informed pig*							
Latency to first bucket (s)	0.21	0.0005	0.09	0.47	0.03	0.56	0.82
Number ofbucket visits	0.82	<0.0001	0.67	0.19	0.17	0.24	0.77
Number of revisits	0.27	0.01	0.33	0.54	0.06	0.78	0.14
Latency to baited bucket (s)	0.27	0.05	0.68	0.27	0.36	0.17	0.95
Proportion of I–NI visits	0.99	–	0.46	–	0.78	–	–
Proportion of closely followed trials	0.98	–	0.34	–	0.86	–	–
Proportion of trials with displacements	0.44	–	<0.001	–	–	–	–

^a^ In phase 2, informed pigs had to find a baited bucket in the search visit and relocate the same baited bucket during the relocation visit, accompanied by a pen-mate that was non-informed about the location of the baited bucket. For informed pigs, performance in relocation visits was compared between phase 1 and 2. For non-informed pigs, performance in search visits was compared between phase 1 and 2

^b^ Pigs were housed pre-weaning either in a multi-suckling (MS) system with 5 sows and their piglets, or with a sow housed in a farrowing crate (FC) and only litter-mates

#### Non-informed pigs

During the paired visits in phase 2, non-informed pigs had a longer latency to visit the first bucket (17.7 ± 1.8 vs. 8.4 ± 2.0 s), visited fewer buckets (2.3 ± 0.2 vs. 4.6 ± 0.3), had fewer revisits (0.03 ± 0.01 vs. 0.22 ± 0.06) and tended to have a shorter latency to reach the baited bucket (55.3 ± 5.5 vs. 71.2 ± 8.4 s) than during the individual visits in phase 1 (Table 3). Moreover, the latency to visit the first bucket and the number of revisits were affected by an interaction between pre-weaning housing treatment and batch. Non-informed MS pigs in batch 2 took the most time to visit the first bucket, compared with non-informed FC pigs in batch 2 and both non-informed MS and FC pigs in batch 1. Non-informed FC pigs in batch 1 had more revisits (numerically in the individual visit) than non-informed MS pigs in batch 1 and FC pigs in batch 2.

#### Interactions between informed and non-informed pigs

There was no difference between the MS and FC pigs in the proportions of I–NI visits (relative to the total number of bucket visits of the non-informed pig), closely followed trials (which occurred in all pens), and trials in which displacements from non-informed pigs towards informed pigs occurred (Table 3). The proportion of trials with displacements was, however, lower in batch 1 than in batch 2. Within MS pairs, there were no trials in which non-informed pigs showed displacement in batch 1. This was significantly different from the proportion of trials with displacements within FC pairs of batch 1 (14 out of 28, *p* < 0.0001) and MS pairs of batch 2 (21 out of 28, *p* < 0.0001). Furthermore, in batch 2, the proportion of trials with displacements was higher within MS pairs than within FC pairs (21 out of 28 vs. 12 out of 28, *p* = 0.03). Informed pigs rarely displaced non-informed pigs; the proportion of trials in which an informed pig displaced a non-informed pig was 0.02 ± 0.01. The proportion of trials in which informed pigs ate from the bait tended to be higher than the proportion of trials in which non-informed pigs ate from the bait (0.79 ± 0.04 vs. 0.47 ± 0.07, *F*_1,15_ = 3.44, *p* < 0.10).

**Fig. 4:**
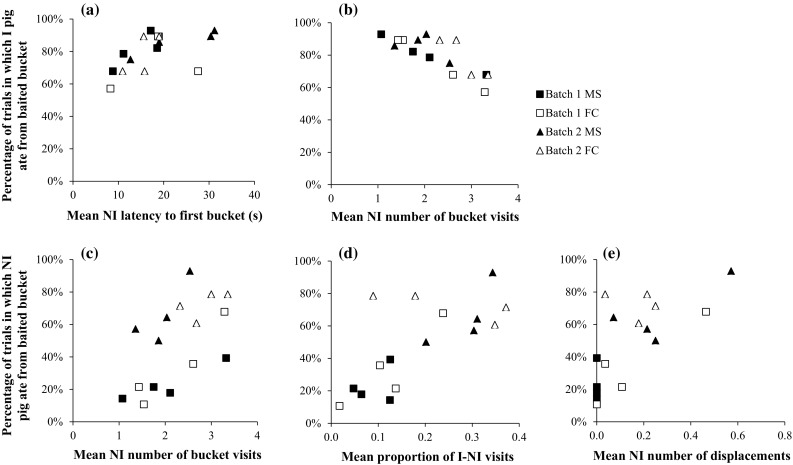
Percentage of trials in which the informed pig (*I*) (**a**, **b**) and non-informed (*NI*) pigs (**c**, **d**, **e**) ate from the baited bucket, plotted against behaviours of the non-informed pigs

Overall, during the first 28 trials of phase 2, the percentage of trials in which the informed pig ate from the baited bucket was positively correlated with the non-informed pigs’ mean latency to visit the first bucket (*r* = 0.62, 14 *df*, *p* = 0.01, Fig. 4a). Moreover, the percentage of trials in which the informed pig ate from the baited bucket was negatively correlated with the mean number of bucket visits per trial by the non-informed pigs (r = −0.73, 14 *df*, p = 0.001, Fig. 4b). Regarding the non-informed pigs, the percentage of trials in which they ate from the baited bucket was positively correlated with (1) the mean number of bucket visits per trial by the non-informed pig (*r* = 0.62, 14 *df*, *p* = 0.01, Fig. 4c), (2) the mean proportion of I–NI visits per trial (*r* = 0.66, 14 *df*, *p* = 0.01, Fig. 4d) and iii) the mean number of displacements by the non-informed pig per trial (*r* = 0.74, 14 *df*, *p* = 0.001, Fig. 4e).

## Discussion

It was hypothesised that pigs reared in an MS system would be better prepared to deal with social and cognitive challenges later in life, because they have experienced more complexity in their physical and social environment than conventionally reared pigs.

### Feed competition test

Several studies have suggested an effect of pre-weaning social environment on aggressive behaviour and hierarchy formation of weaned pigs (e.g. Bohnenkamp et al. 2013; D’Eath 2005; Kanaan et al. 2012; Kutzer et al. 2009; Li and Wang 2011; Martin et al. 2015; Parratt et al. 2006; Verdon et al. 2016). De Jonge et al. (1996) specifically looked at dominance relationships among pigs raised in MS housing and found that submissive pigs hardly displayed aggression towards their dominant pen mate in a post-weaning feed competition test, whereas submissive pigs from barren housing frequently retaliated. Based on this study, it was expected that MS pigs would show less aggression and establish more clear dominance relationships than FC pigs during the FCT, characterised by, for example, less retaliation of the submissive pig and more feed access of the dominant pig. There were, however, no differences between MS and FC pigs in the total frequency of aggression and in the absolute difference in aggression within pairs. The discrepancies in results with the study of De Jonge et al. (1996) may be explained by the larger contrast in pre-weaning conditions in their study (tethered sows in the control group and larger groups of 8 sows and their piglets in the MS system, with outdoor access) combined with a longer exposure time (weaning at 6 weeks of age in their study compared to 4 weeks of age in our study). In pigs, the period of most rapid brain growth occurs until about 6 weeks of age (Dickerson and Dobbing 1967), which may be the most sensitive period for environmental conditions to affect aspects of social and cognitive development. As the MS and FC pigs in our study were already housed under the same conditions between 4 and 6 weeks of age, the post-weaning environment and/or the difference in transition from pre-weaning to post-weaning housing for MS and FC pigs may have partly overruled effects of pre-weaning housing conditions.

Lastly, anecdotally, we noticed some behaviours, which may be related to problem-solving: some pigs walked away from the bucket after not being able to gain access to the feed (7/32 pairs) and subsequently turned to the experimenters (3/32 pairs), e.g. looking at them or standing up against the pen partition with their front legs. Directing attention towards humans in a challenging situation has also been described in other species, such as goats (Nawroth et al. 2016) and dogs (Horn et al. 2012; Passalacqua et al. 2011).

### Informed forager test phase 1

It was hypothesised that, in phase 1 of the IFT, informed MS pigs would learn their task faster and would demonstrate a better working memory than informed FC pigs. Overall, the informed pigs learned their task, indicated by the reduction in bucket visits, revisits, and latency to reach the bait between search and relocation visits. The performance of the informed pigs improved over time, as the latency to reach the baited bucket decreased over the first 9 testing days. These variables were, however, not affected by the pre-weaning housing system. Also, the number of trials to reach the criterion to proceed to phase 2 of the IFT, the number of revisits to buckets (reflecting working memory, see van der Staay et al. 2012), and the percentage of days with a perseverance error were not affected by pre-weaning housing system. This is in contrast to studies in which pigs housed under enriched conditions demonstrated a better short-term and/or long-term memory than pigs housed under barren conditions (Bolhuis et al. 2013; de Jong et al. 2000; Grimberg-Henrici et al. 2016). In these studies, pigs were, however, housed in contrasting environments at the time of the cognitive tests, whereas in our study pigs were housed under the same conditions at the time of testing and had been in those conditions for the 4 weeks preceding the start of phase 1 of the IFT. Similar to the results of the FCT, the timing and duration of exposure to the contrasting environments may have limited the potential effects of pre-weaning housing conditions on the aspects of cognitive performance tested here (also see Bolhuis et al. 2006; Munsterhjelm et al. 2009).

Interestingly, there were some effects of pre-weaning housing treatment on the performance of the non-informed pigs during their search visits. The non-informed MS pigs took more time to complete the test, as their latency to reach the bait was 1.4 times longer, while tending to visit fewer buckets than the non-informed FC pigs. When foraging, pigs are able to use both a win–stay strategy, where a previously baited location should be revisited (Mendl et al. 1997), and a win–shift strategy, where a previously baited location should be avoided (Laughlin and Mendl 2000). A win–shift strategy may have been more frequently reinforced in MS pigs, as the pre-weaning MS system provided multiple locations where feed, foraging materials, and even milk could be obtained, whereas a win–stay strategy would have been more strongly reinforced in the FC system in which resources were available in one fixed location only. Possibly, non-informed MS pigs performed more efficiently, albeit slower, because they had to use only the familiar win–shift strategy. On the other hand, pre-weaning housing treatment effects were not present in the search visits of the informed pigs, possibly because their cognitive abilities were challenged more by the more complex and varied task that they faced, involving the use of a win–stay strategy within a trial and a win–shift strategy between trials. Switching between strategies can reduce pigs’ performance, as this seems to be more difficult than using only one strategy (Laughlin and Mendl 2000). Alternatively, the lower number of bucket visits and longer latency to reach the bait for non-informed MS pigs compared with non-informed FC pigs could also be related to a lower level of general activity and exploration in MS pigs, as Oostindjer et al. (2011b) found an effect of pre-weaning enrichment on the post-weaning expression of these behaviours. Lastly, the longer latency to reach the bait for the non-informed MS pigs compared with the non-informed FC pigs might be related to a potentially lower urgency of responding in a food-related situation, due to the less competitive pre-weaning environment to obtain solid feed.

In short, informed MS and FC pigs learned their task to remember the specific location of a baited bucket after a search visit and a subsequent relocation visit equally well. Non-informed MS pigs, however, searched differently than non-informed FC pigs, with a longer latency to reach the bait, while tending to visit fewer buckets.

### Informed forager test phase 2

During the paired visits in phase 2, informed and non-informed pigs both took more time to reach the first bucket, visited fewer buckets, and had fewer revisits than during the last 8 trials with individual visits in phase 1. This indicates that both informed and non-informed pigs were more hesitant to start visiting buckets in the paired trials, but thereafter searched more efficiently for the bait. The latency to reach the baited bucket did not differ between phase 1 and phase 2 for the informed pigs, but the non-informed pigs tended to reach the baited bucket quicker when they were tested together with the informed pig than when the non-informed pigs searched for the bait alone. This may suggest that the non-informed pigs benefited from visiting the testing arena together with the informed pig, but that this did not necessarily disadvantage the informed pig (also, the proportion of trials in which a pig ate from the baited bucket tended to be higher for informed pigs than for non-informed pigs during paired visits). Potentially disadvantageous effects of the presence of the non-informed pig during paired visits for the success of the informed pig may depend on the non-informed pig’s own investment in searching for the bait, as suggested by (1) the negative correlation between the percentage of trials in which the informed pig ate from the baited bucket and the number of bucket visits by the non-informed pigs, and (2) the positive correlation between the percentage of trials in which the informed pig ate from the baited bucket and the non-informed pigs’ mean latency to visit the first bucket. In addition, for non-informed pigs, both searching independently and following their informed partner may be successful strategies, suggested by the positive correlation between the percentage of trials in which non-informed pigs ate from the baited bucket and (1) the mean number of bucket visits per trial by the non-informed pig, (2) the mean proportion of I–NI visits per trial, and (3) the mean number of displacements by the non-informed pig per trial.

Surprisingly, there were relatively few trials in which the non-informed pig followed the informed pig and subsequently displaced the informed pig from the baited bucket. Overall, we found lower proportions of I–NI visits (0.19 vs. 0.28) and trials with displacements (0.42 vs. 0.65) than reported in the study of Held et al. (2000). This discrepancy may have several explanations. Possibly, the dominance relationship within the IFT pairs was less clear in our study, or changed over time (also see Meese and Ewbank 1972, 1973). To check the latter, the FCT was repeated at the end of batch 2 for the 8 IFT pairs only. All non-informed pigs were still clearly dominant over the informed pigs, with the exception of one MS pair and one FC pair in which dominance was less obvious. Furthermore, Held et al. (2000) used a different feed deprivation method, i.e. restricting daily feed provision to 70% of ad libitum intake, and a different type and quantity of bait, i.e. 400 g of feed. Possibly, this resulted in a higher motivation to perform the IFT than in our study. Also, Held et al. (2000) ran more trials (48–72) in phase 2, although in our study, the proportion of I–NI visits and number of trials with displacements did not increase over time (data not shown), so that running extra trials may not have resulted in more close following of the informed pig by the non-informed pig. Lastly, pigs were tested at a younger age in our study. It has been reported, however, that wild boar piglets and older yearlings have a similar probability of having a certain forager role (Focardi et al. 2015). Hence, the age difference may not have greatly affected the ability of the pigs to adopt a scrounger role and display close following and displacement as part of their foraging strategy.

Thus, both informed and non-informed pigs changed their behaviour in response to being tested pairwise instead of individually. Overall, effects of pre-weaning housing treatment were not distinctly present and partly depended on batch-related differences.

## Conclusions

To conclude, pre-weaning housing in either a complex multi-suckling system or a conventional farrowing system had few distinct effects on pigs’ post-weaning social and cognitive performance, as measured in a feed competition test and an informed forager test. During the feed competition test, familiar pairs of MS and FC pigs showed no difference in the total frequency of aggression and in the absolute difference in aggression within pairs. During individual training in the informed forager test, the learning performance of informed pigs was not affected by pre-weaning housing treatment. During paired visits, both informed and non-informed pigs changed their behaviour in response to being tested pairwise instead of individually. MS and FC pigs, however, showed few distinct behavioural differences during paired visits, and effects of pre-weaning housing treatment were partly batch-dependent.
